# Establishment of a non-bile duct ligation rat model of postoperative pancreatic fistula

**DOI:** 10.1590/acb416526

**Published:** 2026-11-06

**Authors:** Weijie Song, Fan Zhang, Jianfei Tian

**Affiliations:** 1 Tianjin University – School of Pharmaceutical Science and Technology – Tianjin Key Laboratory of Modern Drug Delivery & High-Efficiency – Tianjin – China.; 2 Tianjin Medical University – Cancer Institute & Hospital – Laboratory Animal Center – Tianjin – China.

**Keywords:** Fistula, Models, Animal, Amylases, Lipase, Interleukin-6

## Abstract

**Purpose:**

Postoperative pancreatic fistula (POPF) remains a major complication after pancreatic surgery. We aimed to establish and characterize a simple short-term rat POPF model without bile duct ligation.

**Methods:**

Thirty male Sprague-Dawley rats were assigned to an untreated control group (n = 6) or POPF model group (n = 24). Model animals underwent transection of a major pancreatic duct in the splenic/left pancreatic region with the pancreatic-side stump left open and were evaluated on postoperative days (POD) 1–3 (n = 8/time point). Peritoneal-fluid amylase and lipase, serum interleukin-6 (IL-6), gross abdominal findings, organ inflammation index, and histopathology were assessed.

**Results:**

Model animals developed enzyme-rich peritoneal fluid and marked local inflammation without gross biliary injury. Amylase was 34,316 ± 850; 58,086 ± 900; and 11,800 ± 950 U/L at POD 1–3, respectively; lipase was 10,540 ± 400; 13,057 ± 500; and 4,300 ± 450 U/L. Serum IL-6 increased from 13.0 ± 2.19 ng/L in controls to 126 ± 36, 220 ± 80, and 160 ± 45 ng/L. Overall group differences were significant (all *p* < 0.0001). Histology showed inflammatory-cell infiltration and focal tissue injury.

**Conclusion:**

Major pancreatic duct transection with an open stump produces a reproducible short-term functional rat POPF model and sustained pancreatic leakage through POD3.

## Introduction

Pancreatic cancer surgery has witnessed significant advances in recent decades, reducing perioperative mortality to below 5%. Nevertheless, postoperative mortality remains high, ranging from 16 to 50% in some series^1^. Among the complications, postoperative pancreatic fistula (POPF) is a particularly critical condition that substantially increases morbidity and mortality. POPF is typically defined as the leakage of pancreatic fluid caused by surgical disruption of the main pancreatic duct, leading to severe local and systemic complications^2^. Pancreatic exudates are rich in digestive enzymes that can induce autodigestion, tissue necrosis, and secondary infection^3^.

The International Study Group for Pancreatic Surgery has classified POPF into three categories based on severity and clinical impact^4^. Clinically relevant POPF (CR-POPF) is independently associated with shortened recurrence-free survival and overall survival in patients with pancreatic cancer^5^, highlighting the urgent need for effective prevention and treatment strategies.

Several approaches—including topical hemostatic/sealing agents, pancreatic duct stenting, and pharmacological inhibition of pancreatic secretion—have been explored to mitigate POPF risk^6^. However, results have been inconsistent, and no universally accepted strategy has been established. Development of reliable and reproducible experimental models is therefore crucial for investigating POPF pathogenesis and evaluating novel therapies.

Large animal models using pigs and dogs have been reported^7,8^, but their application is limited due to high costs, regulatory restrictions, and ethical considerations. More recently, Tanaka et al.^9^ established a rat POPF model by transecting secondary pancreatic ducts under indocyanine green guidance. Although innovative, this approach is technically demanding, time-intensive, and the induced pancreatic fistula heals rapidly without intervention, limiting its clinical relevance. Other models involving splenic duct transection combined with splenectomy have also been described^10–12^, but they require complex vascular ligation techniques and fail to generate sustained ascitic leakage.

In the present study, we established a simplified yet robust rat POPF model by transecting the main pancreatic duct at the pancreatic tail with the proximal end left open, thereby simulating persistent leakage of pancreatic juice into the peritoneal cavity. This approach overcomes the technical challenges of duct ligation and provides a stable platform for investigating the pathophysiology and potential therapeutic strategies for POPF.

## Methods

### Ethics statement

Animal studies were conducted under the auspices of the Tianjin Medical University Cancer Institute and Hospital’s Institutional Animal Care and Use Committee (IACUC; Protocol #2025103). All procedures strictly adhered to established institutional protocols for the ethical treatment and humane care of laboratory animals.

### Animals

A total of 30 male Sprague-Dawley rats (approximately 180 g) were obtained from Beijing Vital River Laboratory Animal Technology Co. Animals were housed in individually ventilated cages under standard laboratory conditions (12-h light/dark cycle; temperature 22–26°C; humidity 40–70%) with free access to standard chow and water. Animals were acclimatized for seven days before the experiment.

Animals were allocated to an untreated control group (n = 6) and a POPF model group (n = 24). The 24 model animals were assigned to three predefined endpoint groups: POD1 (n = 8), POD2 (n = 8), and POD3 (n = 8). Untreated controls did not undergo laparotomy or pancreatic duct transection. Control sampling was distributed across the same observation period (two animals at each time-matched point) but, since no postoperative intervention or postoperative state applied to these animals, the six measurements were pooled as one physiological reference group. The smaller number of control animals was consistent with the reduction principle of the 3Rs. No formal *a priori* sample-size calculation was performed because the primary objective was model establishment and short-term characterization.

### Anesthesia and analgesia

For preoperative analgesia, rats were administered a subcutaneous dose of sustained-release meloxicam (1 mg/kg, Boehringer Ingelheim, China) one hour before the surgical procedure. We induced anesthesia using 1–2.5% isoflurane in oxygen within an induction chamber; once lateral recumbency was noted, anesthesia was maintained via a nose cone at a concentration of 0.5–1.5%. To ensure a surgical plane of anesthesia, we continuously monitored the pedal withdrawal and corneal reflexes, alongside a target respiratory rate of 50–70 breaths/min. Body temperature was maintained using a circulating heating pad, and ocular surfaces were protected with lubricant during surgery.

### Surgical procedure

- Animal positioning and preparation. After induction of anesthesia, the rat was placed in the supine position. The abdominal hair was removed, and the skin was prepared aseptically with alternating povidone-iodine and 70% ethanol;
- Abdominal access. A midline laparotomy was performed under sterile conditions to enter the peritoneal cavity;
- Exposure of abdominal organs. The stomach, duodenum, spleen, and diffuse lobulated pancreatic tissue were gently identified. The duodenum and surrounding mesenteric tissues were carefully mobilized to expose the pancreatic ductal anatomy while minimizing manipulation of adjacent vessels and the biliary tract;
- Identification of the target duct. A major pancreatic duct in the splenic/left pancreatic region was identified under direct vision;
- Duct transection. The selected duct was sharply transected using fine microsurgical scissors. The pancreatic-side ductal stump was intentionally left open and unligated to permit pancreatic juice leakage into the peritoneal cavity;
- Inspection. The operative field was inspected to confirm duct disruption and to exclude gross biliary, duodenal, or major vascular injury. No bile duct ligation was performed;
- Repositioning. The pancreas, duodenum, stomach, and surrounding tissues were returned to their anatomical positions. No pancreatic stent, sealing material, or occlusive agent was applied to the transected duct;
- Closure. The abdominal wall was closed in two layers: the fascia with interrupted 4-0 Vicryl sutures, and the skin with 3-0 Vicryl sutures. The operative procedure required approximately 10–15 min per model animal.
- Recovery. Animals were transferred to a warmed recovery area and monitored until spontaneous movement and stable respiration were restored.

The rat pancreas is shown in Fig. 1 as a diffuse, lobulated gland in relation to the stomach, duodenum, and spleen. A major pancreatic duct in the splenic/left pancreatic region is transected, and the pancreatic-side ductal stump is left open and unligated, allowing pancreatic juice to leak into the peritoneal cavity. The ductal system is schematic because substantial anatomical variation exists among rats^10^.

**Figure 1:**
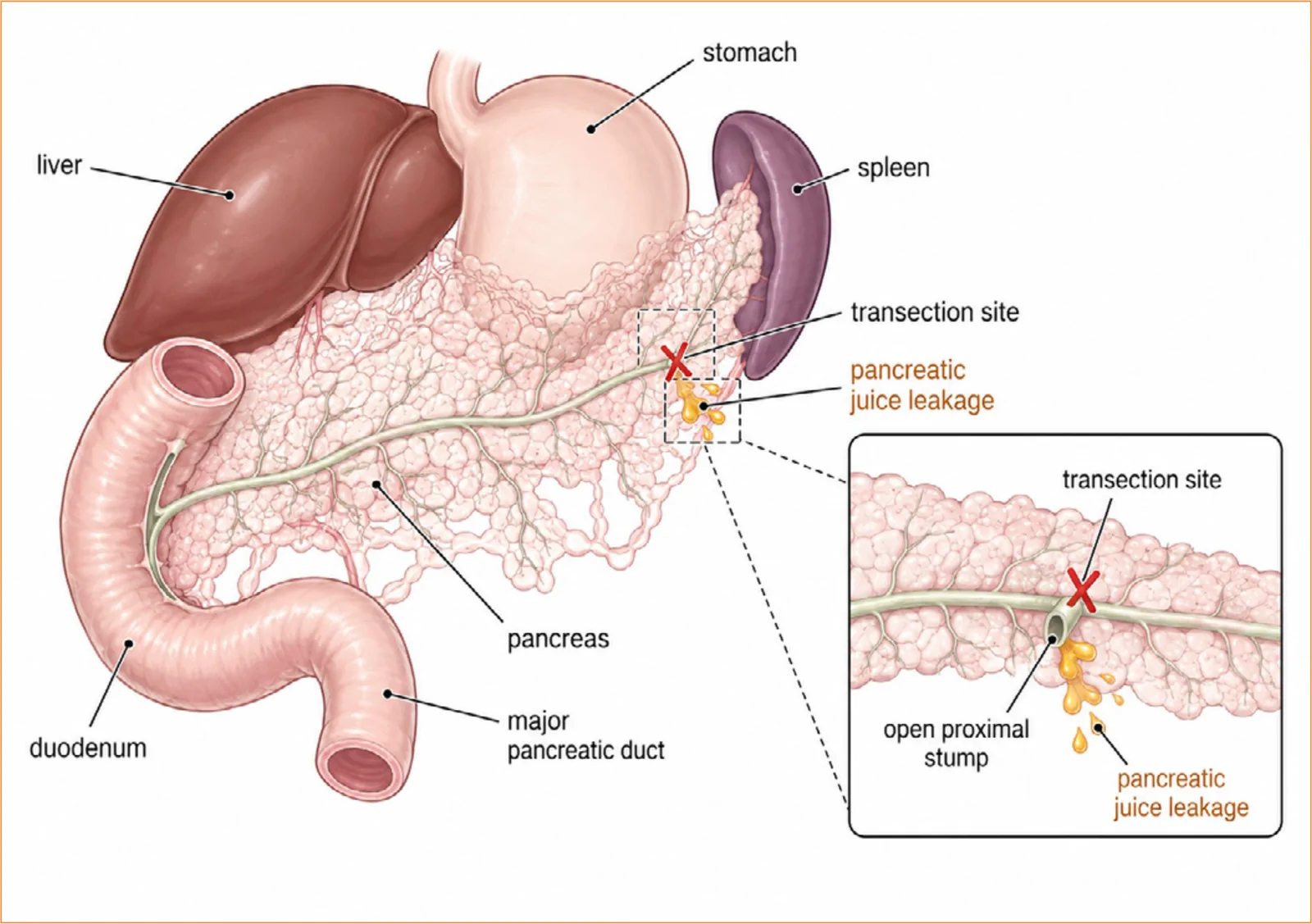
Revised schematic of rat pancreatic anatomy and surgical establishment of the postoperative pancreatic fistula model. The illustration is not drawn to scale.

Source: Elaborated by the authors.

### Postoperative observation and experimental endpoints

Model animals were monitored for survival, general condition, abdominal findings, and postoperative complications until their predefined endpoint (POD1, POD2, or POD3). Because euthanasia was scheduled by study design at these endpoints, survival was not analyzed as a formal time-to-event outcome. At necropsy, the abdominal cavity was examined for peritoneal-fluid accumulation, inflammatory changes, fat saponification, gross biliary leakage, and other complications.

Direct anatomical or mechanical assessment of fistula patency (for example, serial duct-stump photography, cannulation, or contrast injection) was not prospectively performed. Continued leakage was therefore evaluated functionally because of persistent enzyme-rich peritoneal fluid and associated local inflammatory findings at the postoperative endpoints.

### Euthanasia, sample collection, and biochemical analysis

Animals were euthanized at their assigned endpoint using a gradually increasing concentration of CO_2_ in accordance with the approved protocol, and necropsy was performed immediately. Blood was collected for serum interleukin (IL)-6 measurement. Peritoneal fluid was collected for amylase and lipase assays; in untreated controls, the physiological peritoneal fluid available at necropsy was used for the same measurements. Amylase and lipase concentrations were measured using a Mindray BS240VET biochemical analyzer. Serum IL-6 was measured by the laboratory assay used in the original study.

As a secondary macroscopic assessment, the organ inflammation index (OII) was evaluated in a predefined subset of 12 model animals, comprising four animals from each postoperative endpoint group (POD1, POD2, and POD3). The OII was scored from 0 to 3 according to the extent of visible inflammatory involvement in the peritoneal cavity (0 = none, 1 = < 4 mm, 2 = 4–9 mm, 3 = > 10 mm). This subset assessment was used to characterize local inflammatory severity and did not alter the primary biochemical group sizes (n = 8 per postoperative time point).

### Histopathological and immunohistochemical analysis

Pancreatic tissue samples from selected rats were fixed in 10% phosphate-buffered formalin for 48 h, embedded in paraffin, and sectioned at 5 μm. Sections were mounted on positively charged glass slides and stained with hematoxylin and eosin (H&E) for histopathological evaluation by a pathologist. The rat adipose tissue was harvested and cryopreserved. Following cryopreservation, the adipose tissue was evaluated by macroscopic observation to assess the extent of fat saponification, as independently assessed by a board-certified pathologist. Additional sections were subjected to immunohistochemical staining for IL-6 expression.

### Statistical analysis

Data were analyzed using GraphPad Prism 8 (GraphPad Software, San Diego, CA, United States of America) and are presented as mean ± standard deviation (SD). Because unequal variances were observed among groups, overall differences were analyzed using Welch’s one-way analysis of variance (ANOVA), followed by Dunnett’s T3 multiple-comparisons test for post-hoc pairwise comparisons. The OII was a secondary semiquantitative macroscopic endpoint assessed in a predefined subset of model animals (n = 12 total; n = 4 per POD group) and was summarized separately from the four-group biochemical analyses. A two-sided p-value < 0.05 was considered statistically significant.

## Results

Thirty rats were included in the final experimental design: six untreated controls and 24 animals assigned to the POPF model (POD1, POD2, and POD3; n = 8 each). Pancreatic duct transection was technically achieved in the model animals without gross intraoperative biliary injury. At postoperative necropsy, model rats showed visible peritoneal-fluid accumulation accompanied by variable degrees of peritoneal inflammation and fat saponification. Animals were monitored only until their predefined experimental endpoint; no separate survival analysis was performed.

Peritoneal-fluid amylase concentrations were 2,655 ± 151 U/L in untreated controls, 34,316 ± 850 U/L at POD1, 58,086 ± 900 U/L at POD2, and 11,800 ± 950 U/L at POD3. Corresponding peritoneal-fluid lipase concentrations were 4.00 ± 0.75, 10,540 ± 400, 13,057 ± 500, and 4,300 ± 450 U/L, respectively. Serum IL-6 concentrations were 13.0 ± 2.19 ng/L in controls and 126 ± 36, 220 ± 80, and 160 ± 45 ng/L at POD1, POD2, and POD3, respectively.

Welch’s one-way ANOVA demonstrated significant overall differences among the four groups for peritoneal-fluid amylase, peritoneal-fluid lipase, and serum IL-6 (all *p* < 0.0001). Dunnett’s T3 post-hoc testing confirmed marked elevations in all postoperative model groups relative to untreated controls. Amylase and lipase also showed pronounced time-dependent changes across POD1–POD3, with the highest mean values at POD2 and a decline by POD3. Serum IL-6 was numerically the highest at POD2, although variability among postoperative groups was greater than that observed for pancreatic enzymes (Table 1).

**Table 1:** Biochemical and inflammatory characteristics of untreated controls and rats with experimental postoperative pancreatic fistula at different postoperative time points<tfn href="tfn04">@</tfn>.

Parameter	Control (n = 6)	POD1 (n = 8)	POD2 (n = 8)	POD3 (n = 8)	Overall *p* -value
Peritoneal-fluid amylase (U/L)	2,655 ± 151	34,316 ± 850*	58,086 ± 900* #	11,800 ± 950* # $	**1.03 × 10** ^-21^
Peritoneal-fluid lipase (U/L)	4.00 ± 0.75	10,540 ± 400*	13,057 ± 500* #	4,300 ± 450* # $	**1.66 × 10** ^-17^
Serum IL-6 (ng/L)	13.0 ± 2.19	126 ± 36*	220 ± 80*	160 ± 45*	**1.43 × 10** ^-7^

IL-6: interleukin-6;

* *p* < 0.05 *versus* untreated control;

# *p* < 0.05 *versus* POD1;

$ *p* < 0.05 *versus* POD2;

@ Data are mean ± standard deviation.

Amylase and lipase were measured in peritoneal fluid; IL-6 was measured in serum. Welch’s one-way analysis of variance demonstrated significant overall differences among the four groups for all three outcomes (all *p* < 0.0001); Dunnett’s T3 was used for pairwise multiple comparisons. Source: Elaborated by the authors.

Rats with elevated ascitic amylase and lipase levels exhibited pronounced inflammatory responses, accompanied by extensive macroscopic fat saponification in the peritoneal cavity, although the volume of ascites varied among individuals (Fig. 2).

**Figure 2:**
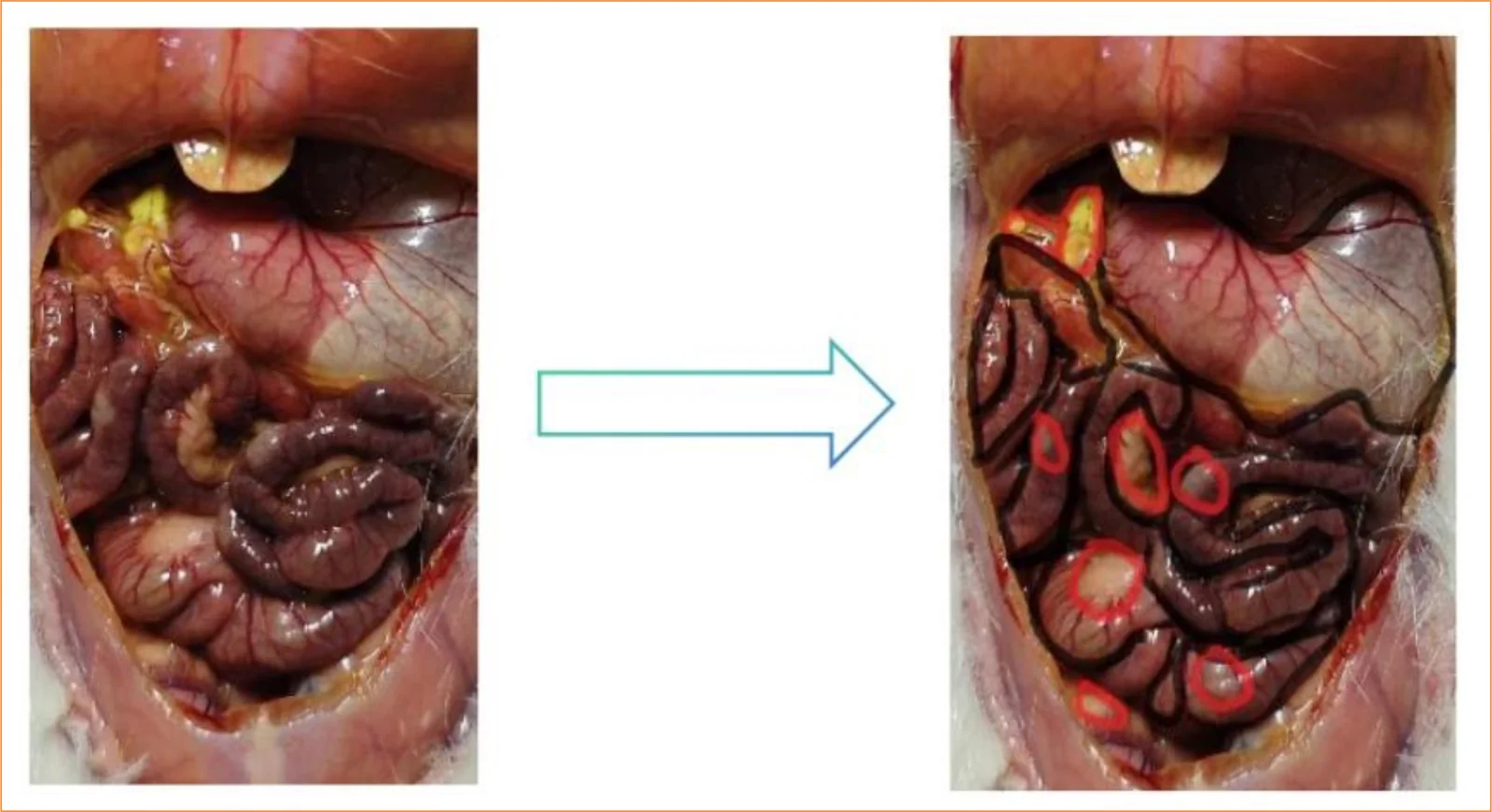
Following the successful establishment of the rat pancreatic fistula model, the abdominal cavity was dissected to expose internal organs. Inflammatory changes in the abdominal organs and fat saponification are observed (highlighted by the red circled area).

Source: Elaborated by the authors.

Analysis of ascitic fluid collected on postoperative days 1–3 showed a marked increase in amylase and lipase concentrations, with a slight decline observed by day 3; these levels remained significantly higher than those of the control group (p < 0.05) (Figs. 3a and 3b). Quantitative assessment of the inflammatory response using the OII further demonstrated a positive correlation between OII scores and ascitic amylase and lipase levels (Figs. 3c and 3d).

**Figure 3:**
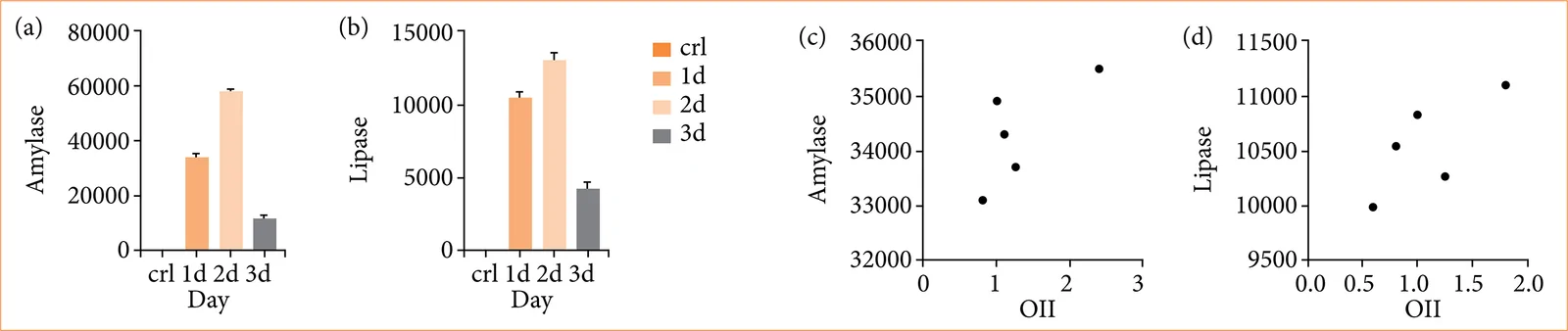
Correlation of ascitic amylase and lipase levels with the organ inflammation index (OII). (a and b) Comparison of serum amylase and lipase levels in the control group with ascitic amylase and lipase levels in the model group, showing significantly higher enzyme concentrations in the model group (*p* < 0.01). (c and d) Correlation analysis between ascitic amylase and lipase levels and OII scores in the model group, demonstrating significant positive correlations (r = 0.7281 and r = 0.7323, respectively).

Source: Elaborated by the authors.

H&E staining showed preserved acinar architecture in untreated controls, whereas pancreatic tissue from model animals showed inflammatory-cell infiltration and focal tissue injury. IL-6 immunoreactivity was low in control pancreatic tissue and increased in model tissue. The pathological findings, together with the persistent enzyme-rich peritoneal fluid, supported successful short-term induction of pancreatic leakage without the need for bile duct ligation.

**Figure 4:**
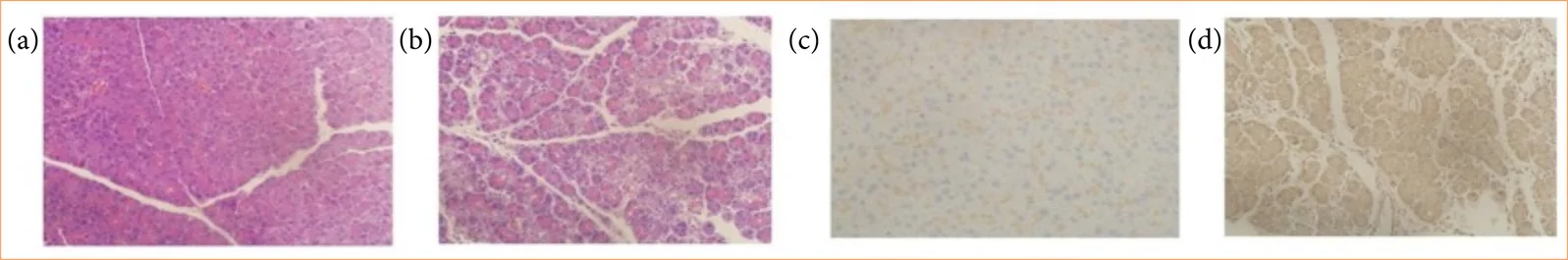
Comparative histological and immunophenotypic evaluation of pancreatic sections. (a) Hematoxylin and eosin (HE)-stained section from the control group, illustrating preserved acinar morphology. (b) In contrast, HE staining of the model group reveals extensive leucocytic infiltration. (c) Basal interleukin (IL)-6 immunoreactivity in control pancreatic tissue, indicating minimal cytokine expression. (d) Enhanced IL-6 expression within the model group, as visualized by immunohistochemical staining.

Source: Elaborated by the authors.

Histological analysis of pancreatic head tissues in the model group revealed pronounced inflammatory infiltration, focal necrosis, and fibrosis at the resection margins, along with peritoneal inflammation, consistent with pathological features of human POPF. Notably, acute pancreatitis was absent (Fig. 4).

## Discussion

POPF remains a major cause of postoperative morbidity and mortality following pancreatic surgery. Despite advances in surgical techniques, the incidence of clinically relevant POPF has not been substantially reduced^10^. The lack of an optimal experimental model has hindered mechanistic investigations and therapeutic development.

Large animal models, such as porcine and canine systems, have been used to mimic POPF^7,8^. While these models are physiologically relevant, their use is limited by high costs, ethical considerations, and technical complexity. The rat, with its well-characterized anatomy, manageable size, and surgical tolerance, offers practical advantages. However, previously described rat models, such as the secondary pancreatic duct transection with indocyanine green guidance reported by Tanaka et al.^9^, are technically demanding and often fail to sustain pancreatic leakage. Moreover, ascitic enzyme levels in these models decline rapidly, which limits their clinical relevance. Other models involving splenic duct transection and splenectomy^10,12,13^ are also technically complex and often fail to produce consistent ascitic collections.

The present study establishes a short-term functional rat model of postoperative pancreatic leakage/POPF using transection of a major pancreatic duct while preserving the biliary tract. The principal features of the model were the development of enzyme-rich peritoneal fluid, gross peritoneal inflammation, and fat saponification, increased systemic IL-6, and inflammatory tissue injury. These findings are consistent with the local and systemic consequences expected after postoperative pancreatic juice leakage.

Our newly developed rat POPF model, based on transection of the main pancreatic duct near the tail with the proximal stump left open, addresses these limitations. It reliably induces persistent leakage of pancreatic juice for at least three days, leading to significantly elevated intraperitoneal amylase and lipase levels, pronounced peritoneal inflammation, and increased IL-6 expression—features that closely mimic the biochemical and histopathological hallmarks of human CR-POPF. The simplified operative approach avoids bile duct ligation, reduces surgical duration (typically < 12 minutes of anesthesia and < 7 minutes of operation), and is reproducible across animals. Because the untreated controls represented one physiological state and did not undergo surgery, their observations were pooled as a single reference group; the smaller control cohort also reduced unnecessary animal use in accordance with the 3Rs.

The OII, developed by adapting the method by Utria et al.^11^, provided an additional semiquantitative macroscopic assessment of local inflammatory severity in a subset of model animals. Four animals from each postoperative model group were assessed (n = 12 total), which was considered appropriate for this secondary descriptive endpoint while limiting additional animal use. Because OII was not a primary efficacy endpoint, these subset observations are interpreted as supportive characterization rather than as a separately powered outcome.

Fistula patency requires careful interpretation. We did not prospectively perform direct anatomical or mechanical testing of the ductal stump using serial photography, cannulation, or contrast imaging. Accordingly, the current study does not claim direct confirmation of anatomical fistula patency. Instead, persistent enzyme-rich peritoneal fluid through POD3 is interpreted as functional evidence consistent with continued pancreatic juice leakage. Future validation should incorporate direct ductal imaging or contrast-based assessment and should extend follow-up beyond the current three-day window.

Despite these limitations, the model has practical advantages. It faithfully replicates the pathophysiological features of human POPF in a small animal system with manageable costs and operational feasibility. It holds promise as a short-term experimental platform for studying the mechanisms of POPF and testing novel local therapeutic interventions, thereby bridging the gap between preclinical research and clinical translation.

## Conclusion

Transection of a major pancreatic duct in the splenic/left pancreatic region with the pancreatic-side stump left open produces a reproducible short-term functional rat model of postoperative pancreatic leakage/POPF without bile duct ligation. It is technically feasible, stable, and reproducible, making it a practical platform for short-term mechanistic investigations. In addition, the model provides a useful preclinical tool for evaluating potential therapeutic interventions, exploring disease progression, and supporting translational research aimed at improving the prevention and treatment of POPF.

## Data Availability

All data sets were generated or analyzed in the current study.
